# A simple, low‐cost method to age mammals? An alternative to cementum annuli analysis

**DOI:** 10.1002/ece3.9710

**Published:** 2023-01-06

**Authors:** Thomas D. Gable, Sean M. Johnson‐Bice, Steve K. Windels

**Affiliations:** ^1^ Department of Fisheries, Wildlife, and Conservation Biology University of Minnesota St. Paul Minnesota USA; ^2^ Department of Biological Sciences University of Manitoba Winnipeg Manitoba Canada; ^3^ Voyageurs National Park International Falls Minnesota USA

**Keywords:** age estimates, beaver dentition, cementum, cementum annuli analysis, mammal dentition

## Abstract

One of the most common and ubiquitous methods to age mammals is by counting the cementum annuli in molars, premolars, incisors, or canines. Despite the ubiquity and perceived simplicity of the method, cementum annuli analysis can be time‐consuming, expensive, inaccurate, and imprecise, and require specialized equipment. Using beavers (*Castor canadensis*) as a test species, we developed a straightforward method to age mammals that requires little specialized equipment. The method consists of: (1) digitizing longitudinally sectioned teeth and measuring the proportion of tooth surface area comprised of cementum (“proportion cementum”), (2) evaluating the relationship between proportion cementum and specimen age (determined from either known‐age samples or cementum annuli analysis), and (3) using the modeled relationship to estimate the age of other individuals based solely on proportion cementum. The relationship between proportion cementum and age was strongly correlated (*R* ^2^ = .97–.98 depending on observer), similar between observers, and similar between known‐age specimens and those aged via cementum annuli analysis. Using this proportion cementum method, two independent observers accurately predicted the age of 80%–84% of specimens within 0.5 year and 96%–98% within 1 year. We suggest this aging method will likely work with most mammal species given the relatively consistent deposition of cementum throughout mammals' lives and has promise to be a simple and quick alternative to cementum annuli analysis regardless of whether one develops proportion cementum models using known‐age specimens or those aged via alternative methods.

## Graphical abstract

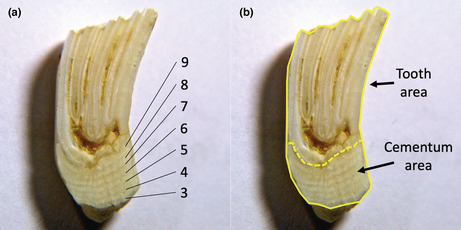

## 1 INTRODUCTION

Determining the age of living beings is crucial for understanding population dynamics (Wikenros et al., 2021), reconstructing past ecological conditions (Lapointe‐Garant et al., 2010), predicting the response of species to changing climatic and environmental conditions (Botsford et al., 2011), and the conservation and management of species and ecosystems (Garshelis, 1990; Morris, 1972). One of the most common and ubiquitous methods to age mammals is by counting the cementum annuli in molars, incisors, or canines (called cementum annuli analysis [CAA]). Cementum is deposited throughout mammals' lives but seasonal differences in diet and nutrition cause a light and dark band of cementum to form each year, which corresponds to periods of increased and decreased cementum deposition (Beasley & Brown, 1992; Grue & Jensen, 1979). As a result, age can be determined by counting the number of cementum annuli in a tooth, although there can be species‐specific differences in the timing and patterns of cementum deposition (e.g., Coy & Garshelis, 1992; Klevezal, 1996; Van Nostrand & Stephenson, 1964).

Aging mammals using CAA is often needed to address fundamental and applied ecological, conservation, and management objectives. This is particularly true for managing and conserving mammal populations that are recreationally hunted or trapped (Garshelis, 1990; Morris, 1972). Many management agencies age teeth from harvested animals via CAA to understand the age structure of harvested animals, the selectivity of hunters, and the reproductive history of individuals (Coy & Garshelis, 1992; Garshelis, 1990). Age structure information is also often incorporated into population dynamics models, which can improve estimates of demographic rates (Plard et al., 2019), predict how populations may respond to environmental change (Koons et al., 2016), and help inform sustainable harvest regulations (Fieberg et al., 2010). Some agencies have dedicated labs or specialists for aging specimens via CAA while many others outsource to private companies specializing in CAA (e.g., Matson's Laboratory which has aged >2.7 million teeth via CAA; https://matsonslab.com). Despite the ubiquity and seeming simplicity of the method, CAA can be time‐consuming (Adams & Blanchong, 2020; Mbizah et al., 2016), expensive (Earle & Kramm, 1980; Mbizah et al., 2016), inaccurate or imprecise (Asmus & Weckerly, 2011; Landon et al., 1998), and require specialized equipment (Brunet‐Rossinni & Wilkinson, 2003). Thus, alternative low‐cost, simple, and rapid methods to age mammals could be extremely beneficial to researchers, conservationists, and managers alike.

We developed a quantitative method to age beavers (*Castor canadensis*), a semi‐aquatic mammal, that is relatively simple, quick, and requires little specialized equipment. The method consists of: (1) digitizing longitudinally sectioned teeth and measuring the proportion of sectioned tooth area comprised of cementum, (2) modeling the relationship between the proportion of sectioned tooth area comprised of cementum and beaver age (determined from either known‐age samples or CAA), and (3) using the modeled relationship to estimate the age of other individuals based solely on the proportion of sectioned tooth area comprised of cementum. We suggest that this simple and quick method will likely work with most mammals given the relatively consistent deposition of cementum throughout most mammals' lives.

## 2 METHODS

### 2.1 Collecting and preparing teeth

We primarily collected teeth from beavers that were legally harvested by local trappers, and the lower mandibles of beavers found opportunistically during fieldwork in the Greater Voyageurs Ecosystem, Minnesota, during 2007–2019. We knew the date of death for most of our specimens because they were killed by trappers. However, for specimens that were collected opportunistically in the field, we estimated month of death based on decomposition of the specimen and time of year.

We extracted the first lower molar (M1) from specimens and longitudinally sectioned them with a Dremel rotary tool. We then used progressively finer grit sandpaper (e.g., 60 grit, then 100 grit, and so on) to polish the sectioned tooth to improve clarity of the cementum. We estimated specimen age by counting cementum annuli (Van Nostrand & Stephenson, 1964). Additionally, a subset (*n* = 16) of trapper‐killed beavers were known‐age specimens that had been live‐captured and ear‐tagged as kits or juveniles in Voyageurs National Park (Windels, 2013), and then legally trapped once they dispersed out of the park.

**FIGURE 1:**
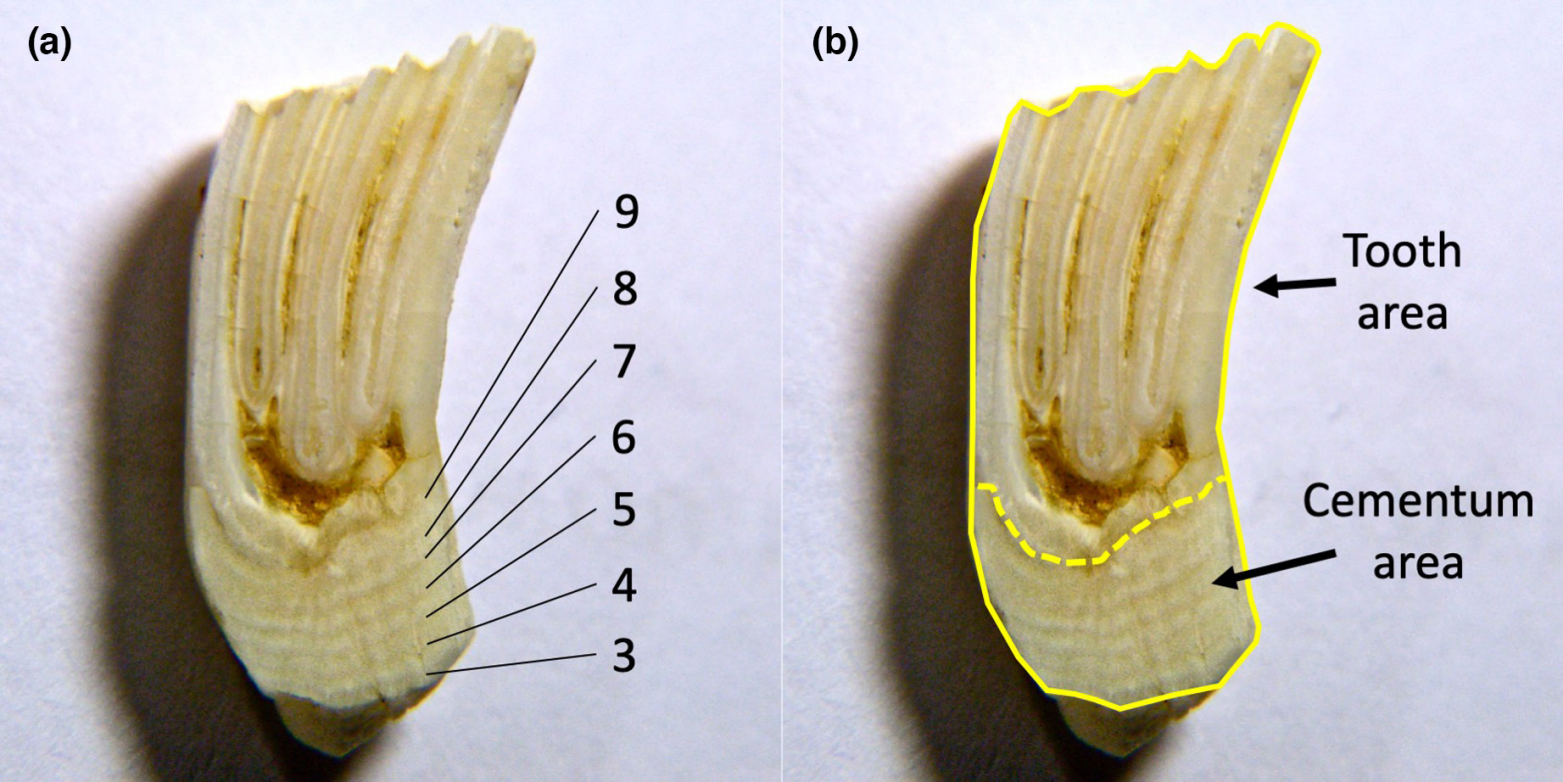
A longitudinally sectioned first lower molar of a 9‐year‐old beaver from the Greater Voyageurs Ecosystem, Minnesota, USA. Panel a shows the cementum annuli used to age the specimen. Panel b shows the digitized surface area of the cementum and the entire tooth. We estimated the proportion of sectioned molars that were cementum by dividing the cementum area by total tooth area.

We photographed longitudinally sectioned teeth using a Nikon D90 with an 18–105 mm lens with macro‐filters, then imported high‐resolution photographs to a computer and counted annuli manually (Figure 1). Beavers do not start depositing cementum around molariform teeth until their third year of life (Van Nostrand & Stephenson, 1964). Thus, to age beavers via CAA, observers must count the number of annuli and then add 2 years. Beavers ≤2 years old can be aged by examining the restriction of the basal cavity of molariform teeth (Larson & Van Nostrand, 1968; Van Nostrand & Stephenson, 1964). We aged beavers to the nearest 0.25 year based on the date of death and a parturition date of May or June (Novak, 1987). In some instances, cementum annuli on the first lower molar were indistinct so we aged the specimen by counting annuli of other molariform teeth under a dissecting microscope.

### 2.2 Measuring cementum

We imported photographs of the longitudinally sectioned molars into ArcGIS 10.2 (ESRI, 2015). We digitized the surface area of the sectioned molar that was comprised of cementum (cementum area) and the total surface area of the molar (molar area; Figure 1). We estimated the proportion of the molar area that was comprised of cementum (referred to hereafter as “proportion cementum”) by dividing cementum area by total molar area. We had two different observers (T. Gable and S. Johnson‐Bice) independently digitize each tooth to evaluate inter‐observer differences, and thus the reproducibility of the results. We then used simple linear regression to model the relationship between proportion cementum and age of all specimens (known age plus those aged via CAA) for each observer. We repeated this process using only known‐age specimens to see if that yielded a different relationship. We compared whether the two observers produced similar results by comparing confidence intervals of the slope and y‐intercept of the models developed for each observer. If confidence intervals of the model developed for each observer overlapped, then we considered the models to have performed similarly.

We then used both observers' model to predict the age of specimens based on the proportion cementum. We estimated the mean error (in years) of the “proportion cementum” model by determining the mean difference between the age of our specimens via CAA and the predicted age from the proportion cementum model developed for each observer. We used R programming software for all statistical tests and assumed α = .05.

## 3 RESULTS

**FIGURE 2:**
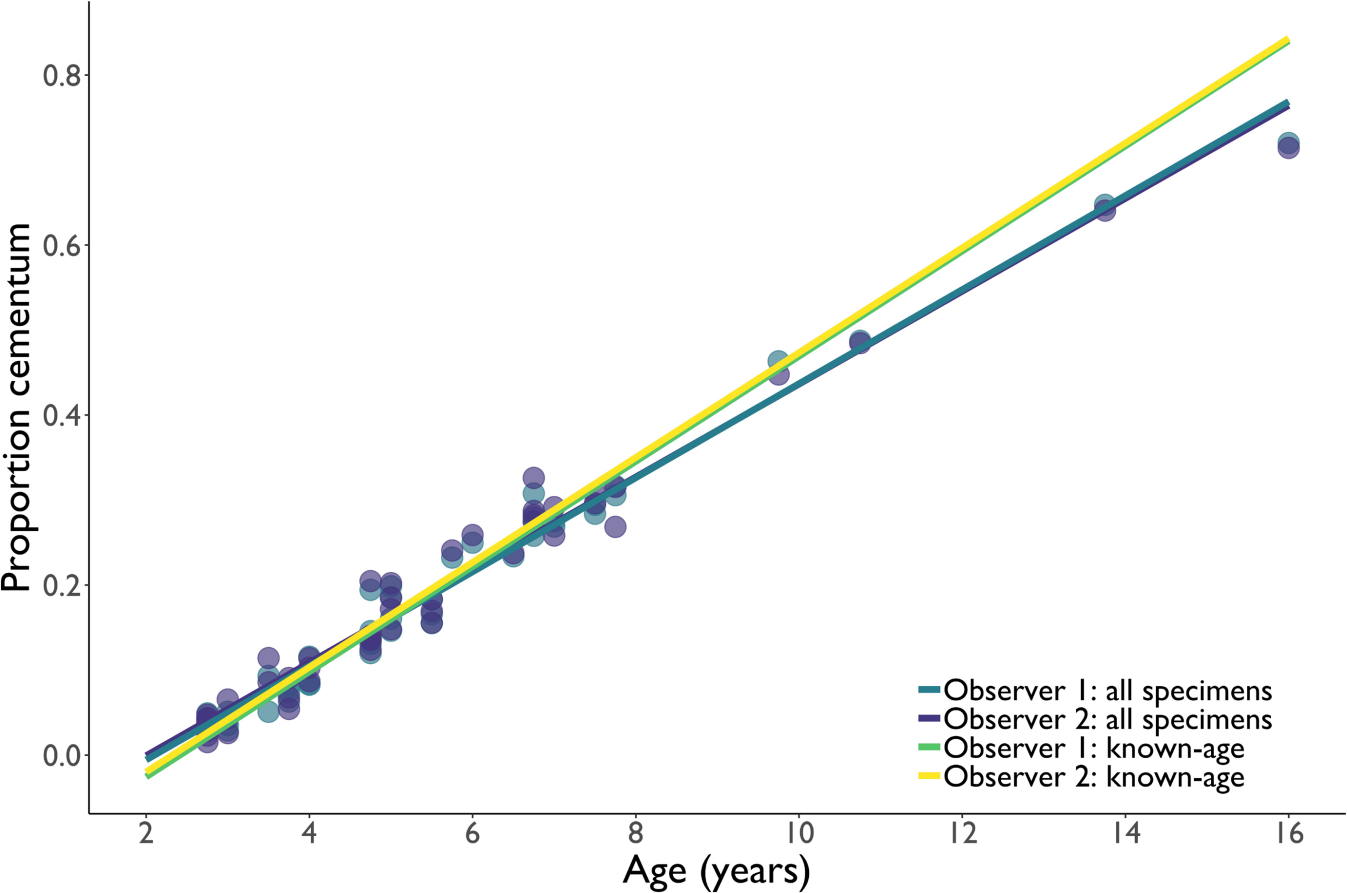
The relationship between the age of beavers and the proportion of cementum visible in the longitudinally sectioned first lower molar from samples collected in the Greater Voyageurs Ecosystem, Minnesota during 2007–2019. Proportion cementum was calculated by dividing the surface area of cementum on a longitudinally sectioned molar by the surface area of the entire sectioned molar. Teeth from 49 beavers were examined by two different observers, and both observers independently calculated the proportion of cementum. Of the 49 specimens examined, 16 were known‐age beavers and 33 were aged via cementum annuli analysis. The blue points correspond to Observer 1 and the purple points to Observer 2.

We used 49 beavers for our analysis, of which 16 were known‐age beavers and 33 were aged via cementum annuli analysis (CAA). We developed a proportion cementum model for each observer using data on all 49 beavers. In doing so, we determined the proportion cementum of the first lower molar was strongly related to age and there was no difference between observers (*R* ^2^ = .98 for Observer 1 and *R* ^2^ = .97 for Observer 2; Figure 2). Furthermore, this relationship remained the same for both observers when using only known‐age specimens (Table 1).

**TABLE 1:** The results of linear regression models, developed for two independent observers, describing the relationship between the age of beavers and the proportion of cementum visible in the longitudinally sectioned first lower molar from samples collected in the Greater Voyageurs Ecosystem, Minnesota, during 2007–2019.

Observer	Model	*R* ^2^	*ß*_1_	95% confidence interval	*ß*_0_	95% confidence interval	Average error (year)	% Aged to within 0.5 year	% Aged to within 1 year
1	All specimens	.98	17.74	17.02–18.45	2.17	1.99–2.33	0.28	84	98
2	All specimens	.97	17.82	16.98–18.68	2.10	1.89–2.31	0.34	80	96
1	Known age	.95	16.17	13.94–18.39	2.41	2.09–2.74	0.33	88	98
2	Known age	.94	16.30	13.87–18.73	2.26	1.89–2.63	0.37	78	94

Proportion cementum models developed using all specimens (known age and those aged via CAA) correctly predicted, depending on observer, the age of 80–84% of all specimens within 0.5 years and 96%–98% within 1 year (Table 1). The average difference between the predicted age via the proportion cementum model and cementum annuli/known age was 0.28 ± 0.25 years (*SD*; range: 0.00–1.06 years) for Observer 1 and 0.34 ± 0.29 years (*SD*; range: 0.00–1.17 years). For known‐age specimens, the proportion cementum model correctly predicted, depending on the observer, the age of 78–88% of specimens within 0.5 years and 94%–98% within 1 yr (Table 1).

## 4 DISCUSSION

We have demonstrated that the proportion of sectioned tooth area comprised of cementum (“proportion cementum”) can predict the age of a mammal species with a high level of accuracy and precision (Figure 2). Although we only examined one species, we think a similar relationship almost certainly exists in other mammal species because cementum is continuously deposited throughout most mammals' lives (Lieberman, 1994), often at relatively similar or predictable rates as mammals age (Grue & Jensen, 1979; Pérez‐Barbería et al., 2020; Tochigi et al., 2019). Proportion cementum is an advantageous metric because it assesses cementum deposition in relation to total tooth area. Using this metric minimizes the influence of variable patterns and rates of cementum deposition around teeth or between individual animals (Earle & Kramm, 1980; Medill et al., 2009), and accounts for differences in the size and shape of the same tooth (Pérez‐Barbería et al., 2020). A similar method developed for red deer (*Cervus elaphus*) found a strong relationship between age and cementum height in the first lower molar after accounting for tooth size, largely because cementum was deposited at a constant annual rate (Pérez‐Barbería et al., 2020).

Ideally, the proportion cementum method (PCM) we describe would be developed using known‐age specimens (Figure 2), which can then be used to age specimens of unknown age. Although the proportion cementum model in our example was linear (Figure 2), models for other populations or species may be non‐linear depending on how cementum deposition changes with age (e.g., cementum annuli width decreasing with age; Tochigi et al., 2019). If a strong relationship between proportion cementum and age of known‐age specimens is identified (e.g., Figure 2), the PCM would likely be a better approach to aging specimens than cementum annuli analysis (CAA). CAA can be prone to error due to observers (Landon et al., 1998; Veiberg et al., 2020), the tooth examined (Asmus & Weckerly, 2011; Moffitt, 1998), reduced clarity of, or indistinct, annuli (Boertje et al., 2015; Earle & Kramm, 1980), irregularity of annuli structure (Rice, 1980), and age of specimens (Asmus & Weckerly, 2011). The PCM avoids many of the challenges associated with CAA because the method does not depend on observing each individual annuli to estimate age. Thus, indistinct, irregular, or false annuli will have minimal effects on age estimates via PCM so long as the cementum can be clearly distinguished from other dental structures (e.g., dentin and enamel). Although delineating the margins of cementum can be subjective in some instances, observer bias appears relatively minor using the PCM (Figure 2). Regardless, we suggest using consistent approaches to delineate cementum when the cementum margins are unclear to minimize variability in proportion cementum estimates.

Collecting a sufficient sample of known‐age specimens is often difficult or unfeasible. In such instances, the most practical implementation of the PCM would be to have a sufficiently large number of teeth aged via CAA, develop a statistical model describing the relationship between proportion cementum and CAA age (ideally using specimens for which the annuli are clear and distinct), and then using the developed model to predict the age of other samples. The PCM is advantageous for researchers or agencies that age specimens frequently (e.g., agencies that age trapped or hunted furbearers and ungulates) because the PCM would reduce the time typically associated with aging specimens. Additionally, almost all tooth specimens, regardless of cementum irregularities or clarity of cementum annuli, could be aged via the PCM.

Proportion cementum models should be species and geographically specific until larger patterns in proportion cementum are identified across species and/or geographical areas. Because patterns in cementum formation and deposition are often species specific, we suspect that most proportion cementum models will be limited to a single species or at best, a few closely related species (e.g., American and Eurasian beavers [*Castor fiber*]). However, we think it is plausible that a proportion cementum model developed for a species in a particular area might perform well for that same species in a different geographical area—especially if the areas are ecologically similar. For example, our proportion cementum model might perform well for beavers in similar southern boreal forest habitats. Our hope is that other researchers will test and evaluate how generalizable the PCM is across taxa (species, genus, and family), environments, and spatial scales.

The PCM has some potential limitations, many of which depend on the life history of the species of interest. In particular, the foraging behavior of species will likely influence how readily the PCM can be implemented. The PCM might be more challenging to implement for species with high tooth fracture rates, tooth loss rates, or uneven rates of tooth wear (e.g., carnivores). In such instances, we recommend selecting types of teeth that are less likely to fracture, be lost, or wear unevenly (e.g., use incisors or molars instead of canines; Van Valkenburgh et al., 2019). On the other hand, using the PCM for species with minimal tooth loss, tooth fractures, and relatively consistent tooth wear throughout life (e.g., herbivores like beavers) will likely be relatively straightforward.

The broad applicability of the PCM may also be limited by species‐specific life history events that cause variations in cementum deposition (e.g., reproduction, illness [Cerrito et al., 2021], nutrition [Lieberman, 1994], or environmental conditions [Lam, 2008]). However, whether variability in cementum deposition from these sources—which often occurs in the microstructure of cementum (Cerrito et al., 2021; Lieberman, 1994)—is large enough to significantly alter the overall relationship between proportion cementum and age is unknown and might be negligible. If this variability is important for larger patterns of cementum deposition, then proportion cementum models could be fine‐tuned to account for important biological (e.g., sex) or environmental factors that can alter cementum deposition in predictable ways (Klevezal, 1996).

We identified several potential practical limitations and challenges associated with the PCM, and although we think these limitations had minimal impact on our results, we think others should be aware of these potential limitations and try to minimize their effect. Although we attempted to longitudinally section each tooth at the halfway point, determining the halfway point of asymmetrical teeth was subjective to an extent. We are not aware of a simple way to standardize this process and instead tried to approximate the halfway point when sectioning teeth. We think sectioning had minimal effect on results given the performance of models. Similarly, digitizing the surface area of cementum can be subjective for a small proportion of samples, especially when the margins of the cementum are indistinct or unclear. Prior experience examining the cementum of the species of interest will likely be helpful in this regard.

In the end, there is no perfect method to estimate the age of mammals. From a practical perspective, we think the PCM is straightforward and allows specimens to be aged in a matter of minutes once a proportion cementum model is developed. Indeed, we can section, photograph, digitize, and age a beaver tooth in 10–15 min without any specialized equipment using the PCM. In contrast, CAA can, at times, be an involved process that requires specialized equipment (e.g., a microtome) to section teeth. As such, many researchers, managers, and conservationists outsource CAA to professional laboratories that often take 1.5–3 months to age specimens (https://matsonslab.com/; https://deerage.com/).

Furthermore, although CAA is the most common method, numerous studies have demonstrated the challenges and limitations of the approach (see above), especially as it pertains to issues of precision and accuracy. For example, CAA can be inaccurate for aging mammals that live in areas with reduced seasonality (e.g., lower latitudes) where annuli are less distinct and thus more challenging to count (Hess et al., 2011; Klevezal, 1996). However, the PCM approach could be a viable solution in such instances so long as the cementum can be easily delineated in the tooth. Indeed, we think the PCM could, where known‐age samples are available, increase the precision and accuracy of age estimates for many mammal species, and in turn, eliminate or reduce many of the typical challenges and drawbacks associated with CAA. Yet, for most species, CAA has remained the best—and sometimes only—tool available for estimating age. Even when known‐age samples are not available, we think, as demonstrated in our study, the PCM could yield similar estimates as CAA. Thus, the PCM has promise to be simple and quick alternative to CAA regardless of whether one uses known‐age specimens or those aged via alternative methods.

## AUTHOR CONTRIBUTIONS

**Thomas D. Gable:** Conceptualization (equal); data curation (lead); formal analysis (lead); methodology (lead); project administration (equal); writing – original draft (lead). **Sean M. Johnson‐Bice:** Data curation (supporting); methodology (supporting); project administration (supporting); validation (lead); writing – review and editing (supporting). **Steven K. Windels:** Conceptualization (supporting); data curation (supporting); formal analysis (supporting); methodology (supporting); project administration (lead); resources (lead); writing – review and editing (supporting).

## FUNDING INFORMATION

There was no specific funding for this project.

## Data Availability

We intend to archive data associated with this manuscript to the Data Repository for University of Minnesota.
